# Therapeutic Implications for Overcoming Radiation Resistance in Cancer Therapy

**DOI:** 10.3390/ijms161125991

**Published:** 2015-11-10

**Authors:** Byeong Mo Kim, Yunkyung Hong, Seunghoon Lee, Pengda Liu, Ji Hong Lim, Yong Heon Lee, Tae Ho Lee, Kyu Tae Chang, Yonggeun Hong

**Affiliations:** 1 Severance Integrative Research Institute for Cerebral & Cardiovascular Diseases (SIRIC), Yonsei University College of Medicine, Seoul 03722, Korea; bkimtwo@gmail.com; 2 Department of Physical Therapy, College of Biomedical Science & Engineering, Inje University, Gimhae 50834, Korea; dangmoo777@naver.com (Y.H.); stormyboy@nate.com (S.L.); 3 Biohealth Products Research Center (BPRC), Inje University, Gimhae 50834, Korea; 4 Ubiquitous Healthcare & Anti-aging Research Center (u-HARC), Inje University, Gimhae 50834, Korea; 5 Department of Pathology, Beth Israel Deaconess Medical Center, Harvard Medical School, Boston, MA 02215, USA; pengdaliu@gmail.com; 6 Department of Biomedical Chemistry, College of Biomedical and Health Science, Konkuk University, Chungju 380-701, Korea; jhlim@kku.ac.kr; 7 Department of Biomedical Laboratory Science, Dongseo University, Busan 617-716, Korea; yhlee@gdsu.dongseo.ac.kr; 8 Division of Gerontology, Department of Medicine, Beth Israel Deaconess Medical Center, Harvard Medical School, Boston, MA 02215, USA; tlee3@bidmc.harvard.edu; 9 National Primate Research Center (NPRC), Korea Research Institute of Bioscience and Biotechnology (KRIBB), Ochang 28116, Korea; 10 Department of Rehabilitation Science, Graduate School of Inje University, Gimhae 50834, Korea

**Keywords:** cancer therapy, ionizing radiation (IR), resistance, cell death, DNA damage, prognostic markers, therapeutic targets

## Abstract

Ionizing radiation (IR), such as X-rays and gamma (γ)-rays, mediates various forms of cancer cell death such as apoptosis, necrosis, autophagy, mitotic catastrophe, and senescence. Among them, apoptosis and mitotic catastrophe are the main mechanisms of IR action. DNA damage and genomic instability contribute to IR-induced cancer cell death. Although IR therapy may be curative in a number of cancer types, the resistance of cancer cells to radiation remains a major therapeutic problem. In this review, we describe the morphological and molecular aspects of various IR-induced types of cell death. We also discuss cytogenetic variations representative of IR-induced DNA damage and genomic instability. Most importantly, we focus on several pathways and their associated marker proteins responsible for cancer resistance and its therapeutic implications in terms of cancer cell death of various types and characteristics. Finally, we propose radiation-sensitization strategies, such as the modification of fractionation, inflammation, and hypoxia and the combined treatment, that can counteract the resistance of tumors to IR.

## 1. Introduction

Radiation therapy is a useful cancer treatment strategy and is a highly cost-effective single-modality treatment. Radiation therapy uses high doses of radiation to destroy or slow tumor growth. Ionizing radiation (IR), such as X-rays and gamma (γ)-rays, is usually used for cancer treatment because it can pass through tissue, break chemical bonds, and remove electrons from atoms to become ionized. The resulting ions can kill or cause serious damage to cancer cells. IR does not kill cancer cells immediately; a significant period of treatment time is required before a large number of cancer cells start to be killed. IR can also lessen or ease problems and symptoms caused by a growing tumor. IR may be given before, during, or after surgery or chemotherapies to enhance the effectiveness of these therapies. IR exposure may be external or internal. External beam radiation therapies, such as X-rays and γ-rays, involve the targeting of radiation at the cancer in a specific part of the body. Internal radiation therapies based on the use of electrons, protons, neutrons, carbon ions, α particles, or β particles are treatments in which a source of radiation, either solid or liquid, is placed inside the body.

The biological effectiveness of IR in killing cancer cells depends on the type of radiation, total dose, fractionation rate, and the targeted organs [1]. Although radiotherapy is an effective cancer treatment, a large portion of patients subsequently experience radioresistance and recurrence of their cancers. Whereas IR alone can be somewhat effective for the treatment of some cancers such as larynx cancer, non-small-cell lung cancer, skin cancer, prostate cancer, cervical cancer, head and neck carcinomas, and lymphomas, it is not effective against other cancers such as breast cancer, bladder cancer, glioblastoma, soft tissue carcinoma, and advanced non-small-cell lung cancer possibly due to the intrinsic radioresistance of cancer cells [2]. IR therapy in combination with other modalities shows promise in the treatment of these radio-resistant cancers. In this review, we will discuss cellular and molecular mechanisms (signaling pathways) of radioresistance in terms of various types of cell death and cytogenetic variations. We will also evaluate strategies to overcome radioresistance during cancer treatment.

## 2. IR-Induced Cell Death Outcomes

**Figure 1:**
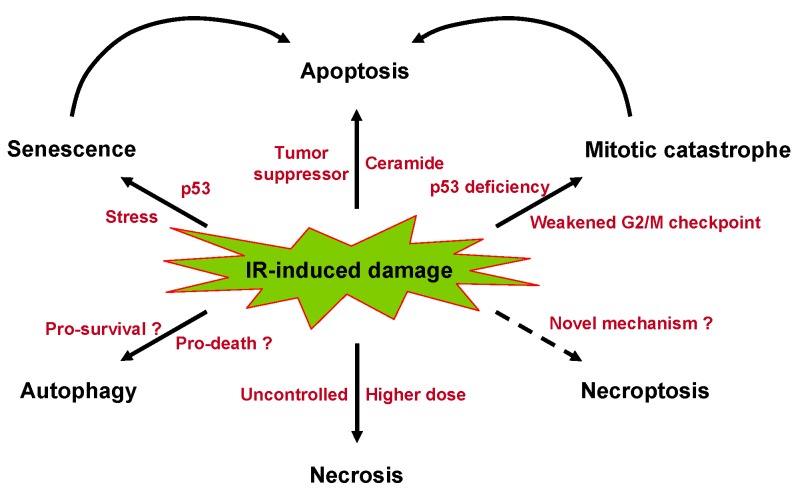
Various modes of cancer cell death induced by IR. Apoptosis and mitotic catastrophe are major forms of cell death induced by IR. Tumor suppressor proteins such as p53 and tumor suppressor lipids such as ceramide promote IR-induced apoptosis. Mitotic catastrophe occurs from premature or inappropriate entry of cells into mitosis caused by irradiation. A p53 mutation can make the cancer cells susceptible to premature mitosis. Furthermore, the loss of the p53-mediated arm of the G2/M checkpoint leads to mitotic catastrophe in cancer cells following exposure to IR. Mitotic catastrophe results from aberrant mitosis and eventually causes cell death through apoptosis or necrosis. Necrosis is an uncontrolled mode of cell death caused by rapid and severe impairment. Even though necrosis is seen less frequently following IR treatment, it does occur after receiving higher doses of IR. Autophagy has a dual role in response to cancer cells to IR; it causes cancer cell survival or death depending on the specific context (pro-survival or pro-death?). Senescence occurs in cancer cells following IR-caused DNA damage and involves p53-mediated cell cycle arrest. Senescent cells later die, typically through apoptosis. Stress-induced premature senescence usually occurs in p53-proficient cells. Necroptosis is a programmed form of necrosis. Although there is evidence that necroptosis is involved in IR-induced cancer cell death, the role of this type of death is a very inchoate area of research (novel mechanism of IR-induced cell death?).

IR therapy, like other types of anticancer treatments, may induce multiple forms of cancer cell death through a variety of molecular mechanisms. Several modes of cell killing, such as apoptosis, necrosis, autophagy, mitotic catastrophe, and senescence, occur after exposure to IR (Figure 1).

### 2.1. Apoptosis

Apoptosis, type I programmed cell death, is a prevalent form of cell death underlying radiation therapy. Apoptosis is characterized by a series of distinctive morphological features including cell shrinkage, membrane blebbing, chromatin condensation, DNA fragmentation, and the resultant formation of apoptotic bodies in which the cellular membrane remains intact. Among a spectrum of cellular components, DNA is the main target of IR. Damaged DNA triggers signal transduction pathways involved in cell cycle arrest and apoptosis. DNA damage monitoring and signaling systems are responsible for cell cycle arrest and checkpoint, and failure of these controls leads to cell death. Second messengers such as ceramide and free radicals can also mediate IR-induced apoptosis [3].

Various factors are involved in IR-induced apoptosis. Poly(ADP-ribose) polymerase (PARP), DNA-dependent protein kinase (DNA-PK), p53, and ataxia-telangiectasia-mutated (ATM) play a role in DNA damage signaling. First, in response to IR-induced DNA damage, especially DNA strand breaks, PARP detects and binds to DNA strand breaks, resulting in quantitative synthesis of a poly(ADP-ribose) chain as a signal for the other DNA-repairing enzymes [4]. A transient and extensive poly(ADP-ribosyl)ation of nuclear proteins occurs early during apoptosis, prior to the commitment of cell death [5]. Several animal studies indicate that PARP is involved in survival responses against IR [6,7,8,9,10]. DNA–PK, a nuclear serine–threonine protein kinase, is a member of the phosphatidylinositol-3 kinase (PI3K) family. The DNA–PK catalytic subunit (DNA–PKcs) forms foci in response to IR and is upregulated in radiation-resistant tumors or in tumors with poor survival rates [11,12]. The deficiency of DNA–PKcs increases IR-induced genomic instability [13]. p53 plays a major role in the cellular response to IR by regulating the transcriptional activation of a number of downstream target genes, such as p21, GADD45, 14-3-3, bax, and Mdm2. ATM functions upstream of p53 by phosphorylating Ser15 and stabilizing p53 [14,15]. ATM is involved in the G1/S cell cycle checkpoint arrest following DNA damage through p53 accumulation and the p53-mediated induction of p21 and, at the G2 checkpoint, through the phosphorylation of the Chk2 protein [14,15,16,17,18]. ATM/ataxia-telangiectasia-related (ATR)-Chk1/2-CDC25 also participates in the G2 checkpoint.

If cellular damage is too severe to be repaired, the cell undergoes apoptosis. Caspase promotes the degradation of key cellular components during apoptosis. Interestingly, caspases also cleave many of the DNA damage repair enzymes to prevent unpromising cellular DNA repair and force apoptosis. A role for the Rb protein in decision-making at the interface between cell cycle and apoptosis has been identified [19,20]. The loss of Rb function leads to increased apoptosis largely by the liberation of the free E2F cell cycle regulator. Hypophosphorylated Rb also triggers apoptosis and then is cleaved by caspases [21,22]. p53-Dependent apoptosis is an important mechanism by which tumor growth and progression is inhibited. The transcriptional induction of Bax appears to be a key feature of this p53-mediated apoptosis [23]. p53 also has a transcription-independent role in apoptosis through its interaction with Bax, which promotes its activation as well as its insertion into the mitochondrial membrane [24,25]. The Bax-independent pathway, which involves protein kinase A, is another mechanism of p53-dependent apoptosis [26]. The roles of p53-independent pathways in IR-induced apoptosis have also been well documented [27,28]. p53-independent apoptosis is generally detected late relative to p53-dependent apoptosis [29]. Interestingly, both p53-dependent and p53-independent apoptotic responses to IR are functionally active in an ATM-independent fashion [30], indicating that apoptosis does not require increased p53 levels. Mdm2, a p53-specific E3 ubiquitin ligase, has been shown to be dispensable for IR-induced apoptosis [31].

Ceramide is a potent mediator of the initiation of IR-induced apoptosis. Several tumor cells resistant to IR-induced apoptosis show defective ceramide signaling [32,33]. A number of downstream targets for ceramide action against IR, such as ceramide-activated protein kinase (CAPK), ceramide-activated protein phosphatase (CAPP), PKC, Bad, Akt, and JNK, have been identified. Pro-apoptotic ceramide signaling has been shown to be mediated by Bad in COS cells [34]. In contrast, PKC activation attenuates ceramide-induced apoptosis in some cancer cell types [35]. Ceramide also suppresses the kinase activity of Akt [36]. Nonetheless, the most important target for ceramide action in IR-induced apoptosis seems to be stress-activated protein kinase (SAPK)/c-Jun N-terminal kinase (JNK). IR utilizes JNK for the amplification of mitochondrial apoptotic death in cancer cells, and JNK activation is PI3K- or reactive oxygen species (ROS)-dependent. Ceramide also induces Fas ligand (FasL) expression, and the soluble FasL-activated sphingomyelin pathway has been demonstrated in IR-induced apoptosis [37].

### 2.2. Necrosis

Necrotic cells are swollen with nuclear vacuolization, protein denaturation/coagulation, and random DNA degradation followed by rupture of cellular membranes. Necrosis has historically been regarded as an uncontrolled, *i.e.*, not genetically regulated, form of cell death. Necrosis is much less common after IR treatment but does occur. The decision as to whether they will undergo apoptosis or necrosis after IR exposure seems to be dose-dependent in some cancer cell types. For example, MG-63 osteosarcoma spheroids die by apoptosis after exposure to 5 Gy IR, while they die by necrosis after exposure to 30 Gy IR [38], suggesting that a much higher dose of IR triggers necrotic cell death.

### 2.3. Autophagy

Cells undergoing autophagy, a form of type II programmed cell death, utilize the autophagic/lysosomal compartment to auto-digest proteins and damaged organelles and to recycle amino and fatty acids. Autophagy is characterized by sequestration of targeted cytoplasmic components and organelles from the rest of the cell within a double-membrane vesicle called the autophagosome. The autophagy pathway is known to be negatively regulated by the PI3K/Akt/mTOR pathway [39]. Hyperactivation of the autophagy pathway contributes to cell death but controlled expression has a pro-survival effect.

Autophagy is a genetically regulated stress response seen in some human cancer cell lines exposed to IR. Compared to apoptosis, autophagic cellular changes are observed after IR in any cell line. Similar to the continuing debate as to whether the induction of autophagy results in cancer suppression or progression, the autophagic response of cancer cells to IR reveals somewhat different effects in terms of radiotherapy. IR treatment induces autophagy in both normal and cancer cells. IR-induced autophagic cell death has been reported in malignant glioma, breast, and prostate cancer cells [40]. Some studies suggest that the induction of autophagy might be an advantageous strategy to increase the anticancer effects of radiotherapy and that chemoagent-induced autophagy provokes sensitization of cells to irradiation and increases the anticancer effects of radiotherapy [41,42]. In particular, the considerable susceptibility of glioblastoma cells to IR-induced cell death is due to autophagy rather than to apoptosis. The inhibition of autophagy also tunes the radiosensitization parameters of malignant glioma cells [43]. On the other hand, autophagy has been found to contribute to the IR resistance of some cancer cell types [44,45]. These conflicting effects might be due to the critical role of autophagy for the removal of misfolded/damaged proteins and organelles. Therefore, autophagy seems to play a double-edged role in the balance between survival and death processes depending on the tissue type and the expression profiles of genes and proteins that regulate the apoptosis machinery.

### 2.4. Mitotic Catastrophe

Mitotic catastrophe or mitotic cell death occurs during aberrant mitosis as a result of aberrant chromosome segregation, leading to formation of giant cells with an aberrant spindle, de-condensed chromatin, and multiple micronuclei. This type of cell death is accompanied by the presence of one or more micronuclei and centrosome overduplication. Together with apoptosis, mitotic catastrophe accounts for the majority of IR-induced cancer cell death. Mitotic catastrophe occurs due to faulty mitosis and causes delayed mitotic-linked cell death that takes place via apoptosis or necrosis. For example, irradiated breast cancer cells die by apoptosis due to delayed cell death associated with mitotic catastrophe after growth arrest [46]. The FasL, TRAIL (TNF-related apoptosis-inducing ligand), and TNF-α death-inducing signaling pathways mediate mitotic catastrophe in these cancer cells through interactions of their ligands and receptors [47]. In this regard, mitotic catastrophe seems to be an absolute requirement for late-type cell death and accounts for the late apoptosis observed after IR exposure.

Interestingly, mitotic catastrophe can be enhanced by p53 deficiency and a weakened G2/M checkpoint [48,49]. The induction of mitotic catastrophe induced by IR is associated with the increased expression of cyclin B1 and the kinase activity of Cdc2 [50]. G2/M checkpoint disruptors, such as caffeine, okadaic acid, or staurosporine, have been shown to promote IR-induced mitotic catastrophe [51]. Mitotic cell death is also enhanced by inactivating mutations in G2/M checkpoint genes, such as p21 and 14-3-3-σ [49,52]. These findings suggest that IR-induced cellular damage may induce the premature entry of cells into mitosis and that mitotic cell death may be a key contributor to the loss of clonogenic potential in tumor cells and solid tumors exposed to IR, especially those with p53 deficiency.

### 2.5. Programmed Necrosis

Recently, a programmed/genetically regulated type of necrosis, necroptosis, was identified. Necroptosis, also called programmed necrosis or type III programmed cell death, is caspase-independent and controlled by the receptor-interacting protein 1 and 3 (RIP1/3) kinases [53]. This type of death is a key process in chronic inflammatory diseases, but its role in cancer is largely unclear. Interestingly, recent studies indicate necroptosis to be a novel mechanism of IR-induced death of some endocrine cancer cell types, such as thyroid and adrenocortical carcinoma cells [54].

### 2.6. Senescence

Senescent cells are viable but non-dividing and undergo irreversible cell cycle arrest, stop DNA synthesis, and become enlarged and flattened with increased granularity. Cellular senescence is a process that results from multiple mechanisms, including telomere shortening, tumor suppressor signals such as p53 and p16INK4A/pRb, and DNA damage. These mechanisms prevent uncontrolled proliferation, and so the cellular senescence can protect cells from developing cancer. Cellular senescence is triggered by a variety of intrinsic and extrinsic stresses, including IR. Senescence can occur in cancer cells following IR-induced DNA damage, eventually leading to cell death mainly by apoptosis. Although p53-independent mechanisms have also been described in IR-induced senescence [55,56], a genetically regulated response to IR-induced DNA damage is usually seen in solid tumor-derived cell lines, especially those with wild-type p53 [57]. IR-induced senescence is a beneficial mechanism through which IR suppresses tumor cell growth. Indeed, the IR-induced retardation of tumor growth is attributable largely to the induction of senescence, not apoptosis, in some lung cancer cell types [58]. Given that IR-induced senescence can usually be achieved at much lower doses of IR than those required to induce apoptosis and that the reduced dose of IR can help prevent adverse side effects of cancer therapy, other strategies using low-dose IR for cancer therapy deserve much consideration.

Stress-induced premature senescence (SIPS) may greatly affect the efficacy of radiotherapy, and the radiation doses achievable using clinical therapeutic regimens can induce SIPS in specific human tumor cell lines. Irradiated cells undergoing SIPS share many cellular and molecular phenomena with cells undergoing replicative senescence. Although replicative senescence is programmed at times when telomeric DNA ends are exposed, SIPS is not programmed but is instead a response to a given stress [59]. Due to the constitutive activation of telomerase, telomeres are typically stable and replicative senescence is not usually induced in cancer cells. However, many anticancer agents, including IR, can induce SIPS in cancer cells while not affecting telomere lengths. These agents produce double-strand breaks (DSBs), and a common cause of SIPS induction in cancer cells appears to be irreparable DNA breaks.

IR-induced DNA damage can occur both directly and indirectly. Actually, the radiation-induced bystander effect in which irradiated cells transfer damage signals to neighboring unirradiated cancer cells is a big part of IR-mediated damage. There is emerging evidence that the bystander effect has a role in the genomic instability and carcinogenesis. [60]. ROS are important factors in the IR-induced bystander effect [61,62]. Additionally, activated macrophages, nitric oxide (NO), and various cytokines have been implicated in this phenomenon [63].

Although cellular senescence can exert a strong tumor-suppressive effect by arresting the growth of cells permanently at risk of malignant transformation, it can also be harmful to the surrounding microenvironment. The senescence-associated secretory phenotype (SASP) is responsible for these deleterious effects, promoting the senescence-associated pro-inflammatory response and resulting tumor progression [64]. Actually, a senescent cell is not a quiescent cell and adopts an immunogenic phenotype, ultimately leading to the SASP. Senescent cells can exert potential effects on their microenvironment by triggering SASP factors such as soluble signaling factors and proteases. Soluble signaling factors, such as interleukins, inflammatory cytokines, and growth and angiogenic factors, are major components of SASP and can affect neighboring cells. In addition, senescent cells also secrete some proteases such as matrix metalloproteinases (MMPs), contributing to tissue remodeling [65]. Moreover, cells undergoing senescence increase the expression of extracellular insoluble molecules such as fibronectin [66]. Fibronectin exerts multiple effects on biological processes, including cell adhesion, survival, growth, differentiation, and migration. Additionally, senescent cells may also exert effects on the tissue microenvironment through the secretion of molecules other than proteins. Together, these SASP components secreted from senescent cells can modify neighboring cells and microenvironment by altering cell-surface receptor signal transduction.

However, SASP is also known to contribute senescence reinforcement in damaged cells. In fact, even a low dose of IR induces SIPS, and these premature cells overexpress several SASP proteins. Some studies suggest that the IR-induced increase in secretory inflammatory signals might contribute to the development of IR-induced damage and premature senescence [67]. Therefore, the secretory program triggered by IR-induced DNA damage can be a critical determinant of the response of tumors to radiotherapy (the sensitivity or resistance to IR) by modifying the tumor microenvironment and its interaction with tumor cells.

## 3. Genomic Instability and Cytogenetic Alterations Induced by IR

**Figure 2:**
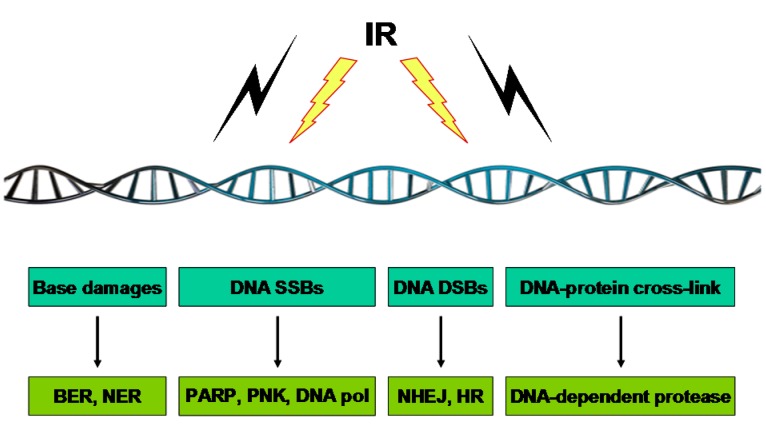
Different types of repairs fix different types of DNA damage caused by IR. Through direct effects involving the deposition of ionizing energy in the DNA itself or indirect effects involving the absorption of ionizing energy by water, leading to the production of water radicals and their subsequent reaction with DNA, IR induces several types of damage to DNA. While IR induces a variety of DNA damages, the DNA DSB is a main lesion responsible for the aimed cancer-cell killing in radiotherapy. DNA repair machineries in response to different types of IR-triggered DNA damage are illustrated.

One of the potent ways in which IR works is through damaging the DNA of exposed tumor tissue, leading to cell death. IR triggers DNA damage in cells both directly and indirectly. The direct effect occurs when the radiation interacts with the DNA molecule itself, while indirect DNA damage is caused by radiolytic products like free radicals that are highly reactive due to the presence of unpaired electrons. IR simultaneously produces various types of lesions, such as base modifications, alkali-labile sites, DNA–DNA intra- and inter-strand crosslinks, DNA–protein crosslinks, single-strand breaks (SSBs), and double-strand breaks (DSBs). These lesions are efficiently detected and repaired by different mechanisms (Figure 2).

### 3.1. Base Damage

IR irradiates and exerts damaging action on free bases, nucleosides, and nucleotides in DNA. IR-induced modifications of bases affect the DNA structure by distorting the DNA double helix. In general, the damaged bases have a minor role in IR-induced cytotoxicity and can be repaired easily through base excision repair (BER) or nucleotide excision repair (NER), with the help of an undamaged template strand and repair enzymes. DNA glycosylases excise the damaged base to generate abasic (AP) sites which are further processed by an AP endonuclease. Although polymerase β is important in the repair of oxidized bases in *in vitro* studies, IR-induced base damage is repaired primarily by the DNA polymerase β-independent long-patch subpathway [68].

### 3.2. DNA SSBs

High-energy IR can disrupt the sugar phosphate backbone, causing either SSBs or DSBs. SSBs are discontinuities or nicks in the deoxyribose backbone of one of the DNA double helixes and are usually accompanied by the loss of a single nucleotide at the site of the break. SSBs arise either directly from damage on the deoxyribose or indirectly as normal intermediates of DNA BER. SSB repair is performed by the serial actions of PARP, polynucleotide kinase (PNK), DNA polymerase, and DNA ligase. XRCC1 also plays an important role in SSB repair by stimulating the activity of PNK at damaged DNA termini [69]. DNA polymerase fills the gap and the remaining nick is then sealed by DNA ligase. Both PARP and XRCC1 mutant cells exhibit an enhanced sensitivity to IR [70,71]. Although DNA polymerase β does not seem to affect radioresistance, it has been shown to contribute to SSB repair through its interaction with XRCC1 [72].

### 3.3. DNA DSBs

DSBs are breaks in the phosphodiester backbone of both strands of the DNA separated by ~10 base pairs or fewer. Unlike SSBs, DSBs are highly toxic, irreparable, and more responsible for a great part of the killing of cancer cells as well as surrounding normal cells because they lead to the large-scale loss or rearrangement of genetic materials during replication and mitosis. Hence, DSBs are the most deleterious lesion produced by IR.

In mammalian cells, DSBs are repaired primarily by the following two mechanisms: non-homologous end-joining (NHEJ) and homologous recombination (HR). The balance between NHEJ and HR is highly regulated, and the choice between these two mechanisms is affected by the chemical complexity of the breaks, chromatin conformation, and the cell cycle.

Simple and primary DSBs are likely repaired by NHEJ. NHEJ starts with the binding of the Ku70/Ku80 heterodimer to the DSB termini, followed by the recruitment and activation of DNA–PK. Incompatible ends are trimmed by nucleases. The ligation complex, which consists of DNA ligase IV, X-ray cross-complementation group 4 (XRCC4), and Xrcc4 like factor (XLF), seals the break. NHEJ is the primary method of repairing breaks due to IR because DSBs produced in euchromatin are repaired mainly by NHEJ throughout the cell cycle [73,74]. HR provides greater repair fidelity than NHEJ [75].

DSBs in heterochromatin are processed mainly by HR mechanisms [76]. In the HR pathway, the MRN (Mre11/RAD50/Nbs1) complex recognizes and binds to DSB ends and subsequently recruits and activates ATM to initiate HR. CtIP (CtBP-interacting protein) is also critical for HR-mediated DSB repair. MRN–CtIP–complex is important for facilitating the DNA resection at the DSB to generate 3’-single-stranded DNA (ssDNA). The ssDNA tail is first coated by replication protein A (RPA), which is subsequently replaced by Rad51 to form a RAD51–ssDNA nucleofilament. This nucleofilament searches for the homologous sequence elsewhere in the genome and mediates DNA strand invasion. RAD51-mediated DNA strand invasion forming a displacement loop (D-loop) can establish a replication fork with a Holiday junction. HR is mostly involved in the repair of clustered and secondary DSBs that occur later after IR during S and G2 phases when the replication fork collapses at unresolved single-strand DNA lesions and the sister chromatids are available to allow recombination processing.

In addition to the formation of radiation-induced prompt DSBs, replication-mediated DSBs are also formed after ionizing radiation [77]. Replication-mediated DSBs, which are chemically distinct from prompt DSBs, are formed when unrepaired non-DSB clustered damage sites meet replication forks to produce replication-mediated DSBs, which require HR for their repair.

### 3.4. DNA–Protein Crosslinks

DNA–protein crosslinks are covalent bonds and biologically active nucleoprotein complexes formed between one strand of DNA and proteins. The crosslinking of DNA to nuclear proteins can impair many cellular processes such as DNA replication, transcription, and repair. DNA–protein crosslinks are induced linearly with γ-rays doses at a frequency of ~150 Gy [78]. At high doses of more than 200 Gy, the number of crosslinks approaches a plateau value corresponding to the number of sites at which DNA attaches to the nuclear matrix [79]. Free-radical formation is believed to be primarily responsible for the production of DNA–protein crosslinks. The role of this crosslinking in response to IR is generally not well defined, although some evidence suggests that it does not play a major role in IR-induced cell killing [80].

## 4. Resistance Pathway

**Figure 3:**
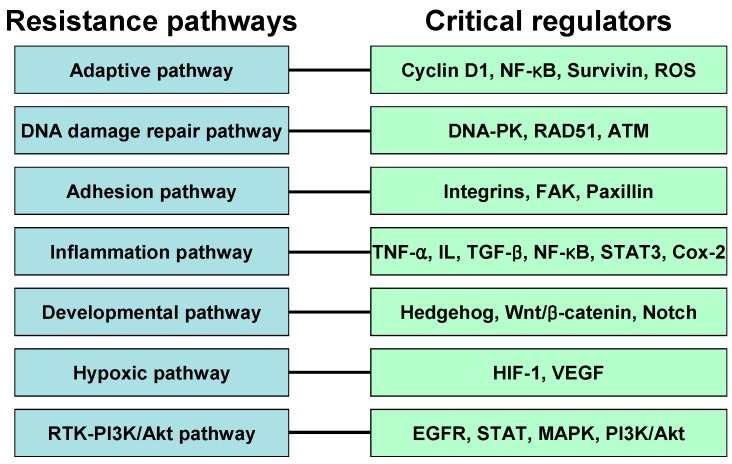
Mechanisms of resistance against radiotherapy. Various signaling pathways may contribute to radiation resistance mechanisms in cancer cells. Resistance against IR may be intrinsic or acquired. The roles of some important regulators in each resistance mechanism have been described.

IR is effective for the treatment of many cancer types; however, in some patients a few tumors become resistant to radiation, making radiotherapy less effective. Resistance to radiation therapy remains a major clinical problem, leading to a poor outcome for cancer patients. Several specific signaling pathways contribute to cellular resistance against IR (Figure 3). Below we review the molecular signaling pathways associated with resistance to IR.

### 4.1. Adaptive Pathway

The recent development of precise radiation delivery techniques increases the efficacy of radiotherapy for cancer treatment. However, when malignant tumor cells recur after radiation treatment, they may acquire a resistance to additional radiotherapy. The use of low-dose IR for radioadaptive protection is known to be efficacious because cells exposed earlier to a lower inducing radiation dose have reduced sensitivity to a higher challenge dose. The exposure of mammalian cells to low-dose IR results in beneficial effects in terms of maintenance of genomic integrity and ability to repair damaged DNA [81,82]. Mitochondria are key sources of ROS as by-products of aerobic metabolism, and induced ROS production is counteracted by mitochondrial enzymatic antioxidant systems. Given that IR treatment significantly upregulates the most powerful antioxidant, manganese-containing superoxide dismutase (Mn–SOD) [83], and that the low-dose IR-induced adaptive response is linked to alterations in the mitochondrial protein import [84], mitochondrial signaling (particularly during apoptosis) seems to be implicated in the adaptive response to IR. As described below, several pro-survival signaling pathways are involved in radioadaptive resistance.

Cyclins bind and activate cyclin-dependent kinases (CDKs), important regulators of the cell cycle. A fluctuation in cyclin expression and the resultant oscillation in CDK activity drives the mitosis. Induced cyclin D1 interacts with CDK4 or CDK6 and activates its kinase activity, which is required for G1/S cell cycle progression [85]. Cyclin D1–CDK4/6 complex has also been shown to phosphorylates and it inactivates tumor suppressor protein Rb whose inhibition induces the expression of several genes involved in S phase progression [86]. This protein is frequently overexpressed in a variety of tumors and contributes to tumor therapy resistance and a poor prognosis in cancer patients [87,88,89]. The enforced translocation of cyclin D1–CDK4/6 from cytosol to nucleus triggers apoptosis [90]. Cyclin D1 also has a function in low-dose IR-induced adaptive radioresistance [91]. Contrary to high-dose IR (e.g., 5-Gy ã-ray) triggering 14-3-3ζ-mediated cyclin D1 nuclear import, low-dose IR (e.g., 10-cGy X-ray) causes a dissociation of the cyclin D1/14-3-3ζ complex and cytosolic accumulation of cyclin D1, indicating that increased cytosolic cyclin D1 expression is required for the low-dose IR-induced adaptive response. Free and increased cyclin D1 protects cells from high-dose IR-induced apoptosis by binding to and inhibiting the action of Bax, suggesting that low-dose IR-induced accumulation of cytosolic cyclin D1 protein plays an important role in suppressing mitochondrial apoptosis in the IR-induced adaptive resistance.

The Rel/NF-kappaB (NF-κB) protein comprises a family of highly regulated dimeric transcription factors that are implicated in the control of diverse cellular functions, such as the stress response, inflammation, cellular growth, and programmed cell death. The high basal NF-κB activity confers the resistance of certain cancers to chemotherapy and radiotherapy [92,93]. Several studies show that the fractional IR triggers NF-κB activation and clonogenic survival [94], indicating that the activation of NF-κB is required for the induction of radioadaptive resistance following exposure to a radiation dose equivalent to that used medically for diagnostic purposes. The IR-mediated induction of cytokines (e.g., TNF-α), MEK/ERK pathways, and ATM is involved in NF-κB activation. IR treatment also induces a variety of NF-κB target proteins that may be responsible for tumor radioresistance. For example, cyclin B1 is responsible for radioadaptive resistance induced by chronic exposure to fractionated IR [95]. A recent study also indicates that the non-canonical NF-κB pathway component RelB regulates Mn-SOD and the resistance to IR of prostate cancer cells [96], suggesting that, as well as the canonical pathway, the non-canonical pathway is important for radiation resistance.

Survivin, a member of the inhibitor of apoptosis (IAP) family, functions to prevent caspase activation and inhibit apoptosis. Survivin has also been implicated in many adaptive responses to cellular stress, acting as an important factor in tumor cell resistance [97]. The low-dose IR induces the elevation of survivin levels and the reduction of apoptosis [97]. The knockdown of survivin induces the loss of the adaptive response, and the survivin-mediated adaptive response has the potential to affect outcomes if induced regularly throughout a course of radiation therapy. A small increase in survival, each day of treatment, induced through exposure to a very low dose of radiation can lead to persistent induction and maintenance of the survivin-mediated adaptive response. Because survivin is overexpressed in malignant cells and can be elevated by both high and very low doses of IR, it is recognized as an important risk factor associated with adverse outcomes in radiotherapy. The survivin-mediated radioadaptive response is dependent upon the ability of cells to activate NF-κB [98].

ROS, which seem to be transiently produced in response to IR, contribute importantly to the cytotoxic effect of IR. Free radicals produced upon IR treatment are the primary source of ROS and reactive nitrogen species (RNS). However, ROS also participate in the cellular signaling processes that lead to an adaptive response that reduces the effectiveness of radiation therapy [99]. ROS generated by IR not only serve to trigger oxidative stress but also contribute to TRAF-mediated NF-κB activation. The NF-κB-mediated anti-apoptotic gene transcription provides enhanced cell survival upon re-exposure of irradiated cells to IR. Antioxidant defense mechanisms, including various antioxidant enzymes, are regulated by NF-κB and prevent tissue damage and ROS-related complications. These antioxidant enzymes, including SOD, catalase, and glutathione peroxidase, have enzymatic ROS-scavenging systems. Peroxiredoxins (Prxs), members of a family of peroxidases, play crucial roles in maintaining redox balance, thereby reducing peroxide-induced redox damage. Peroxiredoxin II (PrxII) is induced by oxidative stress and plays an important role in antioxidant defense by modulating ROS/RNS regulating networks [100,101]. PrxII is significantly upregulated in radioresistant cancer cells, and this upregulation is related to the radioresistant phenotype [102], suggesting that Prx confers radioresistance by eliminating the IR-induced ROS. Therefore, PrxII is likely one of the key players in the protection against oxidative stimuli and in the increased resistance of cancer cells to IR.

### 4.2. DNA Damage Repair Pathway

Although IR is by far the most common therapy for glioblastoma, the most aggressive brain cancer, standard radiotherapy has only limited effectiveness due to its radioresistance. Glioma stem cells confer radioresistance by eliciting DNA damage checkpoint signal transduction and a highly efficient DNA repair system. Deficiency in DNA repair pathways may alter the IR resistance of glioblastomas.

DNA-dependent protein kinase (DNA–PK) plays a central role in the repair of IR-induced DSBs [103], and its deficiency has been implicated in IR sensitivity in glioblastoma cells [104]. DNA-PK, a nuclear serine/threonine protein kinase, phosphorylates several transcription factors and is responsible for DNA repair, cell cycle arrest, apoptosis, and so on. It is a trimeric enzyme consisting of a large catalytic subunit (DNA–PKcs) and heterodimer DNA-binding complex, which comprises 70 (Ku70) and 80 (Ku80) kDa subunits. When a DSB is introduced, the Ku heterodimer binds to the DSB ends, and it then recruits DNA–PKcs. Although the exact mechanism by which the Ku/DNA complex activates the kinase activity of DNA–PKcs is unclear, the conformational change in DNA–PKcs that occurs upon association with the Ku/DNA complex may account for the activation of kinase activity. Because the expression of a kinase-dead mutant of DNA-PKcs fails to confer the radioresistant phenotype in a DNA–PKcs-deficient mammalian cell line [103], the kinase activity of DNA–PK is believed to be needed for DNA repair. In particular, DNA–PK plays a key role in NHEJ and has the structural and regulatory functions of being activated by DSBs and mediating DSB end-joining [105].

In humans, RAD51 acts as a key facilitator of the HR DSB repair pathway [106,107]. Its overexpression is observed in many tumors and is linked to increased radioresistance and poor outcomes [108]. RAD51-related radioresistance is likely to be affected by p53 status because p53 negatively regulates RAD51 expression [108] and is the most frequently mutated gene in human cancers. Several reports suggest that HR and high levels of RAD51 are positively correlated and that RAD51 overexpression increases HR [106,107,108].

PARP is intimately involved in the early stages of DNA damage responses, together with other DNA damage sensors, such as DNA–PK, ATM, and ATR. It recognizes and binds to DNA strand breaks [109] and transfers ADP ribose groups from NAD^+^ to target nuclear proteins, mainly itself [110]. Upon the PAR polymer formations, the poly(ADP-ribose) glycohydrolase (PARG) enzyme catalyzes the hydrolysis of PAR, permitting access of the DNA repair machinery to the lesion [4,111]. PARP has been shown to play a role in both SSB and DSB repair pathways. PARP is a central regulator in BER and participates in SSB repair. Although the mechanisms by which PARP contributes to NHEJ and HR are not as well defined as in BER and SSB repair, PARP has been shown to interact with DSB repair proteins such as NBS1, Mre11, Ku80, DNA–PKcs, and ATM [112,113,114,115]. Given that IR significantly induces PARP and levels of PARP are higher in tumors [116,117], PARP might be useful therapeutic target in radiotherapy. The BRCA1 tumor suppressor protein repairs damaged DNA and, therefore, ensures the stability of the cell’s genetic materials. BRCA1 relocates to DNA damage sites, colocalizes with RAD51, and forms nuclear RAD51 foci in response to IR-induced DNA DSBs [118]. BRCA1 participates in both NHEJ and HR repair process. BRCA1 binds to the MRN complex [119] and suppresses the exonuclease activity of MRE11 [120]. It is also involved in ATM-mediated phosphorylation of NBS1 after DNA damage [121]. The formation of BRCA1-PALB2-BRCA2 complex is essential for RAD51-dependent HR repair [122,123]. Interestingly, potent PARP inhibitors have cytotoxic effects on BRCA1- and BRCA2-deficient cells, including tumor cells [124,125], suggesting that PARP inhibitors can be promising therapeutic agents for cancer with disruption of BRCA genes.

One of the apical activators of DNA damage response is the ATM kinase. Mutation in this gene is associated with ataxia telangiectasia, an autosomal recessive disorder. ATM may regulate radiosensitivity in that ataxia telangiectasia patients are hypersensitive to radiotherapy. The radiosensitizing effects of nonspecific inhibitors, such as caffeine, and highly selective ATM inhibitors, such as KU55933 and CP466722, have also been documented [126].

While IR induces a variety of DNA lesions, including base damage and SSBs, DSBs are widely considered to be the main contributors to IR-induced cell killing through the induction of chromosomal aberrations. Therefore, the pathways for the processing of DSB represent a major mechanism of radiation resistance in tumor cells. The two different cellular pathways contribute to DSB repair and genome integrity in higher eukaryotes: NHEJ, which can be subdivided into DNA–PK-dependent NHEJ, alternative/backup NHEJ, and HR. Normal and cancer cells utilize the DNA–PK-dependent NHEJ pathway extensively to remove DSBs from their genome. Alternative/backup NHEJs, like DNA–PK-dependent NHEJ, are active in all four stages of the cell cycle, but their activity is significantly enhanced during S and G2, probably because the DNA end-resection step occurs primarily in these cell cycle phases [127]. The HR pathway requires sequence information present on intact DNA strands to remove DSBs and to faithfully restore genomic integrity. One form of HR utilizes the sister chromatid as the information donor and is therefore restricted to the late S and G2 phases. Therefore, HR, like alternative NHEJ, regulates DNA damage repair in a cell cycle-dependent manner. The HR pathway is aberrantly expressed in many tumors, and tumor radioresistance, poor prognosis, and increased HR activity are correlated [127], indicating that this pathway is an ideal target for therapeutic intervention in radiotherapy. Therefore, targeting the DNA damage checkpoint response and repair pathways in cancer cells (or cancer stem cells) may offer potential therapeutic advantages to overcome radioresistance.

### 4.3. Adhesion Pathway

Cell adhesion influences cell survival, preventing cell death through multiple mechanisms. The adhesion of cells to the extracellular matrix (ECM) as well as to other cells can regulate various cellular processes, and has been implicated in the development of radioresistance. Especially, cell adhesion to ECM protein fibronectin modulates radiation-induced G2/M arrest and increases resistance to IR. Adhesion, migration, and invasion of the surrounding ECM by cancer cells are mediated predominantly by cell surface receptors, including integrins and other associated adhesion molecules.

Integrins are transmembrane receptors that are the bridges for the interactions between cell and cell or cell and ECM, and are involved in cell adhesion in many cellular processes, including immune defense, embryogenesis, wound healing, and metastasis. Resistance to radiotherapy is modulated by the interaction between the ECM and integrins. Integrins modulate the cellular response to IR and decrease IR-induced cancer cell death [128,129]. Some studies indicate that integrins and type 1 insulin-like growth factor receptor (IGF-1R) may play a concerted role in the radioresistance of cancer cells [130,131,132,133]. Integrins are heterodimeric proteins comprising á and β subunits, and at least 18 á and 8 β subunits have been described in mammals. Among the various integrin units, β_1_ integrins are associated with very late antigen receptors and conjoin with the á_3_ subunit to create the á3β1 complex, which reacts to netrin-1 and reelin. β_1_ integrins, whose expression is tightly regulated by IGF-1R, are believed to mediate resistance to IR through inhibition of JNK activation [133]. Likewise, β_1_ downregulation or inhibition plays an important role in increasing cancer cell sensitivity to radiotherapy [133], implying a therapeutic strategy. However, the role of JNK activation in radioresistance is somewhat controversial given that β_1_-mediated signaling through the FAK/cortactin/JNK pathway was recently reported to be associated with radioresistance in head and neck squamous cell carcinoma [134]. Integrin cleavage can also be a sensitizing factor to IR in prostate cancer [135].

Integrins regulate cellular functions through the signaling molecules colocalized in the focal adhesion complex. Focal adhesions and large macromolecular assemblies form structural links between the ECM and the actin cytoskeleton and are important sites for regulatory signal transduction. Focal adhesion kinase (FAK) is a cytoplasmic focal complex-associated tyrosine kinase that is elevated in a variety of human cancers [136]. It triggers the expression of various antiapoptotic proteins. Compared to HL-60 cells, HL-60/FAK cells are highly resistant to IR-induced apoptosis through the activation of the PI3K-Akt pathway and the induction of IAP proteins such as c-IAP-2 and XIAP [137]. FAK overexpression usually prevents IR-induced mitochondria-dependent apoptosis. Paxillin, one of the main focal adhesion proteins of integrin signaling, is a multi-domain adaptor protein that is localized in the focal adhesion complex [138]. To facilitate adhesion, paxillin is phosphorylated and activated by FAK [139]. Mechanistically, interactions of the β_1_ integrin with Akt, p130Cas, and paxillin contribute to radiation resistance [140]. Furthermore, the interconnected cytoskeletal network involving ECM, integrins, cytoskeleton, nuclear matrix, and chromatin organization may affect the cancer cell responses to various anticancer agents, including IR.

### 4.4. Inflammation Pathway

The immune system plays a pivotal role in controlling tumor development, suppression, or progression. Radiotherapy can modulate antitumor immune responses, modifying the tumor and its microenvironment through the activation of cytokine cascades. Cytokines, such as TNF-α, IL-1α, IL-1β, IL-6, and TGF-β, are produced by tumor cells and tumor-infiltrating lymphocytes and can greatly influence cellular radiosensitivity and the onset of tissue complications. For example, IL-6 upregulation is positively linked to radiation resistance while its inhibition enhances the radiation sensitivity of prostate cancer cells [141]. The balance between proinflammatory and anti-inflammatory cytokines is critical in determining the outcome of, and adverse reactions and resistance to, radiation treatment. Cytokines can influence the dose-dependent IR response by their pleiotropic effects, modulating inflammation, invasiveness, and fibrosis. Many factors, including radiation dose, tissue type, and the intrinsic characteristics of tumor cells, can cause the local response to be a pro- or anti-tumor effect after radiation exposure. Total dose and number of fraction applied are likely to be key determinants in the immune response after radiation exposure. A large single dose of irradiation may produce a more potent immune response than low-dose fractionated radiation. An inflammatory IR response can also favor the invasion of cancer cells, providing a favorable environment for tumor promotion and metastasis. The ability of radiation to increase invasiveness has been reported for pancreatic, rectal, and colon cancer cells [142]. Both IL-8 and IL-6 are involved in the IR inflammatory response and enhance cancer cell invasiveness [142,143]. In addition, IR-induced IL-1β expression can also favor cancer cell invasion [142]. Moreover, some preclinical models suggest that radiation-activated TGF-β can contribute to metastasis, inducing the appearance of mesenchymal characteristics [144].

Radiation-induced transcription factors, such as the NF-κB family and signal transducer and activator of transcription-3 (STAT3), are linked to radioresistance due to the production of a variety of proteins including cyclin D1, VEGF, MMP, and proinflammatory cytokines [145]. Whereas the induced expression of these target genes in response to radiotherapy is responsible for the inducible and/or adaptive radioresistance, constitutively activated NF-κB or STAT3 contributes to intrinsic radioresistance [145]. Upregulation of NF-κB by TNF exposure confers increased radiation resistance to cancer cells. The STAT3-mediated radioresistant phenotype is the result of multiple mechanisms in combination with radiation-induced activation of the Janus-activated kinase (JAK)-STAT pathway [145,146,147]. Erythropoietin, which plays an important role in the neoplastic transformation and malignant phenotype progression observed in malignancy, induces resistance to IR in human cancer cells in a JAK2-dependent manner [148].

Radiation is also known to induce inflammation through cyclooxygenase-2 (Cox-2) [142]. Cox is necessary for the conversion of arachidonic acid to the family of prostaglandins and its activity in cancer can be directly stimulated by NF-κB after radiation exposure or indirectly by the activity of several cytokines. Cox-2 is overexpressed in various types of cancer [142]. In particular, Cox-2 upregulation is associated with a higher tumor grade and distant metastases in breast cancers [149]. This protein has assumed an important role as a therapeutic target for anticancer and anti-inflammatory therapies. In particular, Cox-2-derived prostaglandins are thought to protect cells from radiation-induced damage, contributing to tumor growth and resistance to radiation therapy. Cox-2 activation via the PI3K/AKt pathway can also induce resistance to radiation in human cancer cells [150]. Collectively, the use of specific inhibitors or drugs that manipulate cytokine pathways should be encouraged to improve radiation therapy because TNF-α, IL-1β, IL-8, IL-6, or TGF-β can influence the response to IR by inducing inflammation, cancer cell invasiveness, and fibrosis in irradiated tissues.

### 4.5. Developmental Pathway

Although IR represents the optimum non-invasive therapy for many cancers with benefits in terms of overall survival, it may also cause therapy failure due to the presence of cancer stem cells. Cancer stem cells account for only a small part of the bulk tumor, but are the cardinal reason for therapeutic resistance. Developmental pathways, including the Hedgehog, Wnt/β-catenin, and Notch pathways, have long been postulated to play an important role in maintaining cancer stem cells and thereby the cancer itself. Self-renewal pathways are known to contribute to cancer stem cell resistance to IR. IR treatment commonly induces stemness in cancer cells.

The Hedgehog signaling pathway plays a crucial role in development and patterning during mammalian embryogenesis [151,152,153]. The binding of the Hedgehog ligand to the Patched receptor regulates target genes that are involved in many cellular functions including survival, proliferation, and metastasis. This pathway also affects the maintenance of the cancer stem cell population by exerting pro-proliferative effects in cancer stem cell/stem cell-like populations in many types of cancer [154,155,156]. Moreover, Hedgehog pathway signaling is upregulated in various cancers [157,158,159] and is associated with the epithelial-to-mesenchymal transition (EMT) [160,161]. Given that stem cells confer resistance to chemotherapy and radiotherapy, the Hedgehog pathway may have a role in radiotherapy. For example, this pathway modulates radiation therapy resistance in head and neck cancer [162]. The combination of IR and the Hedgehog pathway inhibitor cyclopamine demonstrates improved tumor cell control both *in vitro* and *in vivo* [162], suggesting an additive benefit over either therapy alone.

In fractionated IR treatment, a small amount of radiation is delivered to the patient at regular intervals over a prolonged period. However, the first fraction of radiation can stimulate the migratory and invasive capacities of the tumor cells, resulting in tumor relapse and metastasis [163,164]. IR treatment can activate multiple signaling pathways in tumor cells, which modulate cellular functions and induce the secretion of cytokines and growth factors, promoting the migration and invasion of various types of cancer cells, including glioblastoma, the most common and lethal brain cancer [163]. The Wnt/β-catenin pathway, especially the canonical pathway, is believed to be important in maintaining the stemness of cancer stem cells and in promoting cellular invasiveness through the regulation of the EMT in many cancers [165]. The Wnt/β-catenin pathway mediates the radioresistance of glioblastoma by promoting invasion [166,167], raising the interesting possibility that invasiveness and radioresistance are phenotypes associated with Wnt/β-catenin signaling. Therefore, targeting radiation-induced invasion can be a promising therapeutic modality for the treatment of cancer.

The Notch signaling pathway plays crucial roles in the control of cell–cell communication, cell fate, and pattern formation [168,169]. The Notch pathway is also involved in stem cell proliferation, differentiation, and apoptosis [170]. The role of the Notch pathway can be either oncogenic or tumor-suppressive in that it functions as an oncoprotein in most human cancers, including lung, colon, cervical, prostate, pancreatic, and head and neck cancer, but acts as a tumor suppressor in skin cancer, hepatoma, and small-cell lung cancer (SCLC) [171,172,173]. This pathway is often overactivated in a variety of cancers [174], and is believed to be a candidate target for therapies designed to eliminate cancer stem cells. Notch activity in established breast cancer cells is increased in response to radiotherapy [175]. High Notch activity is correlated with radioresistance and poor prognosis in non-small-cell lung cancer (NSCLC) patients [176]. Moreover, cancer stem cells lose their ability to repopulate the tumor after IR treatment when Notch activity is suppressed.

### 4.6. Hypoxia Pathway

The variety and severity of DNA damage from IR is influenced by the tumor microenvironment. In particular, the molecular oxygen status of a tumor is known to influence the biological effect of IR; cells become radioresistant under hypoxic conditions [177,178]. Many tumors comprise poorly oxygenated hypoxic regions that are radioresistant and this depletion of oxygen results in inefficient formation of DNA strand breaks by IR. Moreover, patients displaying highly hypoxic tumors have a much poorer outcome than those with well-oxygenated tumors.

Hypoxia inducible factor 1 (HIF-1), the critical regulator of cellular responses to hypoxia, has been shown to be involved in hypoxia-related tumor radioresistance [179,180,181]. HIF-1 modulates the expression of over 100 genes involved in the regulation of proliferation, apoptosis, and angiogenesis. HIF-1 expression levels increase under IR treatment as early as 24 h, and this effect persists for up to one week. After irradiation, when radiosensitive cells are killed and they free up oxygen to be delivered to the previously hypoxic regions, the hypoxic cells undergo reoxygenation. The oxidative stress generated during reoxygenation causes activation of the HIF-1 pathway. Therefore, paradoxically, reoxygenation causes activation of the HIF-1 pathway, which usually responds to low oxygen levels. Due to its critical role in tumor radioresistance, HIF-1 may constitute a therapeutic target for hypoxic tumors.

The interactions among hypoxia, angiogenesis, and radioresistance are well known. Activation of the PI3K/Akt pathway, an important pathway involved in radioresistance, contributes to the increased transcription and expression of HIF-1 [182]. VEGF, a major transcription target of HIF-1 in hypoxic conditions, regulates various endothelial cell functions and induces tumor neovascularization [183,184]. One of the important mechanisms by which inhibition of the epidermal growth factor receptor (EGFR) enhances tumor oxygenation is the regulation of VEGF. Cooperation between the PTEN mutation and EGFR activation upregulates VEGF even under normoxic conditions through the PI3K/Akt pathway. Tumor cell proliferation and angiogenesis, which may be implicated in radioresistance, require the interaction between EGFR and VEGF and downstream signaling of the PI3K/Akt pathway.

### 4.7. RTK-PI3K-Akt Pathway

In cancer cells, radiation-induced or constitutive signaling pathways that act through receptor tyrosine kinases (RTKs) are implicated in modulating radioresistance. EGFR, an ErbB family member, is a membrane-spanning tyrosine kinase receptor that plays an important role in regulating tumor cell proliferation, survival, invasion, and angiogenesis. High-level EGFR expression is correlated with radioresistance and poor outcome after radiotherapy in preclinical and clinical studies [185,186]. EGFR exerts prosurvival and pro-proliferation activities through the activation of downstream signaling pathways, including the PI3K/Akt, STAT, and Ras–Raf–MAPK [187,188,189]. Among them, the PI3K/Akt pathway is a key downstream survival pathway activated in cancer cells. The Akt serine/threonine protein kinase, a central kinase downstream of PI3K, is involved in different aspects of cellular regulation, such as cell growth, proliferation, survival, migration/invasion, metastasis, angiogenesis, and metabolism.

Similar to ligand stimulation, IR stimulates EGFR signaling into its downstream PI3K/Akt [190,191,192]. Hyperactive PI3K/Akt signaling is one of the factors responsible for the development of cancer cells with increased resistance to radiotherapy. The role of PI3K/Akt activity in radioresistance has been reported in various cancers, including those of the brain, lung, colon, cervix, and head and neck. For example, constitutive phosphorylations of Akt, presumably at Ser473 and Thr308, mediate radiotherapy resistance in NSCLC cells [193], and activation of the PI3K/Akt/Cox-2 pathway enhances resistance to radiation in human cervical cancer [150]. RLIP76 can regulate PI3K/Akt signaling and induce radioresistance in pancreatic cancer, which is an aggressive malignancy with characteristic resistance to conventional chemo-radiotherapy. The function of the PI3K/Akt pathway in response to radiation is believed to be independent of p53 status. Moreover, although PI3K activates other prosurvival effectors, such as SGK, Akt plays a central role in PI3K-mediated radioresistance. Given that inhibition of Akt activity leads to impaired DNA DSB repair following radiation and enhanced radiosensitivity, activation of Akt improves post-irradiation cell survival by promoting the repair of IR-induced DNA DSBs, and might be of particular importance in terms of radiotherapy outcome. Akt seems also to be involved in the radioresistance of cancer stem cells through an as-yet-unknown mechanism.

Like apoptosis, autophagy has also been recognized as an important factor in determining the response of tumor cells to radiotherapy. The PI3K/Akt signaling pathway is a major regulator of autophagy [194,195]. Autophagy exhibits a biphasic effect against exposure of tumor cells to IR, inducing a cytoprotective or cytotoxic effect [196,197]. The PI3K/Akt/mTOR pathway has been implicated in the cytotoxic effect of radiation-induced autophagy. Akt inhibition exerts a radiosensitizing effect in malignant glioma cells by inducing autophagy [198].

As mentioned above, two pathways are involved in DNA DSB repair: the NHEJ and HR pathways. In the NHEJ repair pathway, Akt forms a functional complex with DNA-PKcs at the DNA DSB site and stimulates DNA–PKcs kinase activity, all of which are necessary for the DNA DSB repair. Akt-dependent phosphorylation/activation of DNA-PKcs indicates that Akt is involved in the first initiating step of DNA DSB repair [199]. Akt can also be activated by the MRE11–RAD50–NBS1 (MRN)–ATM pathway as well as the PI3K pathway. The role of Akt in DSB repair is further substantiated by radiation-induced colocalization of γH2AX foci and phosphorylated Akt in the site of DSB [199,200,201]. BRCA1, BRCA2, and RAD51 are critical components of the HR repair pathway, to which Akt activity is also linked. Particularly, whereas Akt stimulates the NHEJ pathway, its aberrant activation suppresses the HR pathway and generates genetic instability in tumor cells [202]. Because NHEJ is the predominant pathway in mammals, activation of the EGFR/PI3K/Akt signaling pathway is crucial for tumor cell survival after radiation and the inhibition of Akt activity by targeting the RTK–PI3K–Akt axis suppresses faithful repair of IR-induced DNA DSBs and improves radiotherapy efficacy [203,204,205,206].

## 5. Strategies for Overcoming Resistance to IR

**Figure 4:**
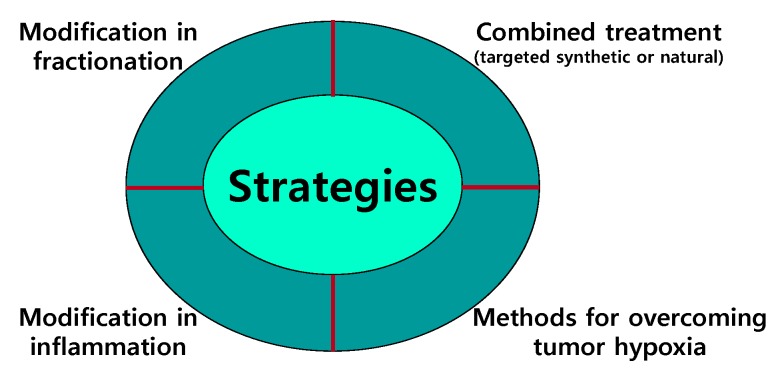
Strategies for overcoming radioresistance in cancer treatment. Several therapeutic strategies have been suggested to overcome the radioresistance of cancer cells.

Radiation-induced apoptosis is a major cell death form in tumors derived from hematopoietic, lymphoid, and germ cells. However, epithelial solid tumors show extensive resistance to IR-induced apoptosis. Radioresistance is a serious concern, causing radiotherapy failure and subsequent tumor relapse. Therefore, novel therapeutic radiosensitizers are urgently needed for overcoming tumor radioresistance and thus improving the outcome of radiotherapy. A growing number of inhibitors that target specific components of radioresistance pathways are being developed for clinical use. Moreover, natural radiosensitizers have also been developed in an effort to compensate for the limitations of synthetic inhibitors. Several strategies have been suggested for overcoming resistance to IR in cancer treatment (Figure 4).

### 5.1. Modifications in Fractionation

Fractionated radiation therapy for cancer has advantages over a single administration of radiation because it can increase the anticancer therapeutic effect and decrease the occurrence of severe side effects in normal tissues. In the most conventional fractionated radiation therapy, about 2.0 Gy of radiation is given per day, about 10 Gy per week, and up to about 60 Gy for six weeks in total. Unfortunately, however, this strategy cannot sufficiently control locally advanced cancers and is not satisfactory to patients due to its limited efficacy and the occurrence of side effects. New strategies such as hypofractionation have been proposed to compensate for the limitations of conventional fractionated radiation therapy. First, clinical trials are assessing the administration of larger doses of radiotherapy per fraction in fewer fractions. Therefore, in the future, conventional radiotherapy strategies will be replaced largely by hypofractionated radiation therapy, which delivers very potent, focused beams of radiation to tumors but gives fewer radiation treatments. Hypofractionation involves the delivery of daily doses larger than 2.0 Gy, shorter overall treatment times, and lower overall doses. In this hypofractionated radiotherapy, although the dose of the single fraction is higher, the total dose delivered is lower than in conventional radiotherapy. Hypofractionation regimens take advantage of greater cell killing per fraction of the cell survival curve and combat accelerated repopulation of tumor tissues. In particular, the use of hypofractionated radiation treatment to specifically target brain tumors will be beneficial in terms of saving normal brain tissue and preserving the brain’s various sub-functions (such as memory) compared to irradiating the entire brain. Researchers are investigating various ways of giving standard external beam radiotherapy, such as hyperfractionation and accelerated fractionation as well as hypofractionation. In hyperfractionation, a smaller dose of radiation (e.g., ~1.2 Gy) is delivered twice per day with an interval of about 6 h, allowing the total dose of radiation to be increased, which has therapeutic benefits and fewer side effects. In accelerated fractionation, a relatively high dose of radiation (e.g., ~1.6 Gy) is applied twice per day but the total dose is identical to that used in conventional radiotherapy. Because the total duration is shorter than that of conventional fractionation treatment, this treatment strategy is advantageous, especially in very rapidly growing tumors.

### 5.2. Combined Treatment

In an effort to overcome tumor radioresistance, much research has focused on developing tumor-specific radiosensitizers. Combined anticancer therapy has been emerging as an established clinical practice. In many solid tumors, simultaneous treatment with chemotherapeutic agents and radiation is far more effective than sequential single treatment. This is based on the principle that a single chemotherapeutic or radiotherapeutic agent has low therapeutic activity in many tumor types, whereas combining therapeutic agents with unrelated action mechanisms may result in synergistic, or at least additive, anti-neoplastic effects.

#### 5.2.1. Selective Molecularly Targeted Synthetic Agents

Hence, a variety of radiosensitizing agents have been developed to target DNA DSB repair pathways, including small interfering RNAs, aptamers, antisense RNAs, and small-molecule inhibitors, which are being clinically developed by a number of corporations. Various inhibitors of Akt, mTOR, and Chk1/2 have been shown to act as potential radiosensitizers [198,203,207,208,209,210,211,212].

Because an intact or hyperactive HR is often correlated with radioresistance, exploring means of inhibiting the HR repair pathway in cancer cells may be beneficial. A number of radiosensitizers have been found to inhibit HR. Nucleoside and base analogs such as gemcitabine, TAS-106, gimeracil, pentoxifylline, and caffeine are examples [127]. Furthermore, the ATR inhibitor VE-821 and the Chk1/2 inhibitor AZD7762 have also been reported to inhibit HR [213,214]. The tyrosine kinase inhibitors imatinib and erlotinib, the HDAC inhibitor PCI-24781, the proteasome inhibitor MG132, and the HSP90 inhibitor 17-allylamino-17-demethoxygeldanamycin (17-AAG), all of which are less directly implicated in the DNA damage signaling, are also suggested to radiosensitize cancer cells by targeting the HR repair pathway [127].

Emerging evidence shows a novel role for radiotherapy as a therapeutic partner of cancer immunotherapy. IR can act as an immune adjuvant, contributing to systemic antitumor immunity. IR leads to the activation of several immunological proteins and transcription factors that modulate the expression of numerous immune mediators that may promote cancer development. Thus, the targeting of IR-induced inflammatory signaling pathways offers the opportunity to improve the clinical outcomes of radiation therapy by enhancing radiosensitivity. Several studies have reported the radiosensitizing effect of NF-κB inhibition in various models [215,216,217]. Clinical approaches for NF-κB inhibition to induce tumor radiosensitization include corticosteroids, phytochemicals, proteasome inhibitors, and synthetic peptides. The inhibition of Cox-2, one of the central enzymes in the inflammatory response, using pharmacological inhibitors such as ascoxibs, celecoxib, and SC-236, represents a radiosensitization strategy [142]. The combined treatment of radiation and cisplatin might complement the intrinsic inability of this drug to induce redistribution of calreticulin, a cisplatin-binding protein, thus affecting cell death. The combination of radiation and the poly(ADP-ribose) polymerase inhibitor veliparib exerts its effect by promoting tumor immunogenicity [218]. Preclinical studies have demonstrated that combining radiotherapy with immune stimulation can induce antitumor immunity, enhancing cell death.

#### 5.2.2. Radiosensitizers Derived from Natural Products

The clinical benefits of synthetic inhibitors in terms of improving treatment outcomes are limited. In addition, the unintended and adverse side effects of these inhibitors are growing concerns. These limitations prompt the development of novel and more effective radiosensitizers to address these unmet medical needs. Naturally occurring radiosensitizers are frequently believed to be relatively safe compared to synthetic compounds because they are normally found in foods. Moreover, natural products with antioxidant and immune-enhancing effects are believed to have better biological and radioprotective effects for normal cells. Several natural radiosensitizers have been reported (Table 1).

##### Curcumin

Curcumin, a polyphenol, is a natural compound derived from tumeric and curry powders. Curcumin has various anticancer activities. It suppresses cancer cell proliferation, downregulates NF-κB target genes, reduces the activity of growth factor receptors, and counteracts tumorigenesis [219]. Interestingly, curcumin suppresses all three stages of carcinogenesis: initiation, promotion, and progression. Resistance to radiation can be caused by increased expression of NF-κB-induced prosurvival genes such as antiapoptotic Bcl-2 family members. Curcumin has been shown to downregulate STAT3 phosphorylation as well as NF-κB expression induced by various inflammatory stimuli [220]. Some studies show that curcumin sensitizes cancer cells to IR through the inhibition of NF-κB activation [221,222]. Curcumin has also been reported to confer protective effects against radiation-induced toxicity in normal cells/tissues through the activation of Nrf2 antioxidant signaling pathways [222,223,224]. The fact that curcumin can achieve efficient radiosensitizing effects without any toxicity makes its development as an adjunct to standard radiotherapy an important goal.

**Table 1:** Effective natural radiosensitizers for the treatment of cancer. These compounds act as radiosensitizers for cancer cells and, at the same time, radioprotectors for normal cells.

Name	Types	Source	Radiosensitization	Target in Radiosensitization	Radioprotection	Target in Radioprotection
Curcumin	Polyphenol	Tumeric	Arrest, Apoptosis	NF-kB	Yes	NRF2, Antioxidant enzymes
Resveratrol	Polyphenol	Grapes	Apoptosis, Senescence	NF-kB, Cox-2, 5-LOX	Yes	Not determined
Genistein	Polyphenol	Soybean	Arrest, Apoptosis	Akt, Erk, Survivin, Cycline B, NF-kB	Yes	Not determined
Quercetin	Polyphenol	Ubiquitous	Arrest, Apoptosis	ATM	Yes	Not determined

##### Resveratrol

Resveratrol is a polyphenol compound found in various dietary sources, including grapes, wine, soy, berries, and peanuts. Resveratrol, a natural antioxidant, exerts an anticancer effect by suppressing the proliferation of a variety of cancer cells. Resveratrol has been shown to potentiate the apoptotic effects of IR as well as cytokines and chemotherapeutic agents [222,225,226]. Resveratrol induces radiosensitization through the inhibition of NF-κB, Cox-2, and 5-lipooxygenase (5-LOX) [222]. Resveratrol also potentiates the IR-induced accumulation of ceramide, a potential anticancer agent, by promoting its *de novo* biosynthesis [227]. Interestingly, resveratrol treatment enhances IR-induced premature senescence rather than apoptosis in NSCLC [228].

In addition, chromosome aberration analyses in an irradiated mouse model support the use of resveratrol as a radioprotector that has the potential for widespread application [229].

##### Genistein

Genistein is a naturally occurring soybean-derived isoflavone glycoside that inhibits protein tyrosine kinase. Genistein can inhibit cancer cell growth by inducing apoptosis and differentiation and/or by suppressing angiogenesis and metastasis. Anticancer effects of genistein are mediated by the inhibition of protein tyrosine kinases, topoisomerase I/II, protein histidine kinases, and the expression of cell cycle–related genes in multiple malignant tissues. Genistein in combination with IR may enhance the efficacy of radiotherapy in numerous cancer cells by inhibiting DNA synthesis, cell division, and cell growth. For example, genistein greatly enhances the radiosensitivity of human esophageal cancer cells by suppressing radiation-induced activation of survival signals such as Akt and Erk [230]. Combination treatment with genistein and IR significantly induces G2/M arrest followed by apoptosis by reducing the expression of survivin and cyclin B in cervical cancer cells [231]. Genistein also increases radiation-induced apoptosis and promotes arrest at the G2 phase of the cell cycle in leukemia cells [232]. Because cells in the G2/M phase are more sensitive to radiation damage than those in other phases, G2/M arrest in response to genistein treatment can sensitize cancer cells to IR-induced death. Combined treatment-induced G2/M arrest has been also reported in other cancer cell types, such as prostate cancer cells [233]. In contrast, genistein can also protect from IR-induced injury in human normal lymphocytes [234]. Moreover, the administration of genistein alleviates the IR-induced injury in mice without adverse effects on motor activity, body weight, or histopathology [224]. NF-κB inhibition mediated by genistein is also suggested to be an underlying mechanism by which genistein exerts its anticancer and radiosensitizing effects. The increased cancer cell death induced by the combined treatment of genistein and IR is assumed to occur largely via the inhibition of NF-κB, leading to the decrease in cyclin B levels and/or the increase in the levels of the cdk inhibitor p21, thus promoting G2/M arrest and increasing radiosensitivity.

##### Quercetin

Quercetin, one of the main components of flavonoids, is distributed ubiquitously in vegetables and fruits such as apples, nuts, berries, onions, cauliflowers, and cabbages, and in red wine, tea, and the propolis of honeybee hives. It has multiple effects on the immune system and has been found to have antioxidant, antiviral, anti-inflammatory, and cardioprotective activities. Quercetin has anticancer activity against several types of cancer by promoting apoptotic cell death and inducing cell cycle arrest [235,236,237]. Quercetin also induces autophagy [236]. Its radiosensitizing activity has been demonstrated in various cancer cells and a human tumor xenograft model [238]. Mechanistically, quercetin enhances tumor radiosensitization through inhibition of the ATM-mediated pathway, one of the key DNA damage signaling pathways, in response to radiation [238]. Quercetin exerts radioprotective and immunoprotective effects against radiotherapy in normal tissue [239,240]. It also effectively protects lung tissue against radiation-induced pulmonary injuries [240].

### 5.3. Modification of Inflammation

Recently, radiation oncologists came to pay attention to tumor stroma to increase the efficacy of radiotherapy. Depletion of tumor-associated macrophages can increase the antitumor effects of IR. For example, VEGF-neutralizing antibodies can enhance the antitumor response to IR through downregulation of IR-induced VEGF in macrophages [241]. Immunestrategy exerts synergistic antitumor activity with local radiation in preclinical studies. Since radiotherapy elicits *in situ* vaccination, some novel approaches have tested radiotherapy in combination with cancer vaccines or adoptive T-cell transfer [242]. Antibodies targeting immune checkpoint receptors and/or inducible T-cell costimulatory receptors can also be successfully treated with radiotherapy [242].

### 5.4. Methods of Overcoming Tumor Hypoxia

Tumor hypoxia is considered one of major problems for radiation therapy because hypoxic tumor cells are more likely to survive IR. To overcome tumor hypoxia, several therapeutic strategies have been developed. The most common methods employed to overcome tumor hypoxia-related resistance include radiation fractionation, the use of high linear energy transfer (LET) particles in the management of hypoxic tumors, and the implementation of bioreductive drugs, such as adjuvant treatment. Several methods of overcoming tumor hypoxia are outlined below.

First, the direct delivery of sufficient oxygen to locally advanced solid tumors during IR treatment is one of the simplest and most effective options. Indeed, hyperbaric oxygenation, red blood cell transfusions, and erythropoietin administration have been investigated as methods of increasing tumor oxygenation. Although these approaches showed therapeutic benefits in preclinical studies, they are not in widespread use because of the conflicting results of clinical trials.

In the 1970s, nitroimidazole derivatives, such as misonidazole, were found to mimic the effect of oxygen in the radiochemical process, raising the possibility of their use to enhance the cytotoxic effect of IR under hypoxic conditions [181,243]. However, dose-limiting toxicities, mainly due to its low solubility, led to a limited therapeutic benefit of misonidazole in clinical trials. Effective doses of this drug were found to cause peripheral neuropathy, which has prevented its routine clinical use. To solve this problem, other nitroimidazole derivatives with high solubility, such as etanidazole and doranidazole, have been developed as promising candidates [181].

Because of its active role in the radioresistance of hypoxic tumor cells, HIF-1 has been recognized as a prime molecular target for sensitization to the therapeutic effect of radiation. For example, targeting tumor metabolism through HIF-1 inhibition enhances the radiation response in cervical and head and neck xenograft tumors [244]. The well-known HIF-1 inhibitor YC-1 was found to downregulate HIF-1α and HIF-2α through post-translational modifications and to inactivate the carboxyl-terminal transactivation domain of HIF-1α [245]. It was also reported to decrease HIF-1 target gene expression under hypoxic conditions, thereby inhibiting the growth and spread of tumors [246,247]. Inhibition of the radiation-induced upregulation of HIF-1 activity by YC-1 dramatically suppresses tumor recurrence after radiotherapy [248]. Inhibition of the dimerization of HIF-1α with HIF-1β also likely exerts a radiosensitizing effect because dimerization is a prerequisite for DNA binding by, and transcriptional activity of, HIF-1 [249]. In this sense, acriflavine may also be a potent HIF-1 inhibitor because it binds directly to HIF-1α and inhibits HIF-1 dimerization and transcriptional activity [250]. However, the radiosensitizing activity of acriflavine has not been examined to date. The negative regulation of key factors that upregulate the expression or activity of HIF-1 represents another approach to HIF-1 inhibition. The PI3K-Akt-mTOR signaling pathway is known to upregulate the expression of HIF-1α protein. Therefore, inhibitors of these pathways, such as RAD-001, LY294002, wortmannin, and rapamycin, contribute to radiosensitization presumably by suppressing HIF-1. HSP90 can also enhance HIF-1 expression; it binds to HIF-1α in competition with RACK1 and inhibits the oxygen-independent degradation of HIF-1α [251]. Additionally, 17-AAG or deguelin, a novel naturally occurring inhibitor of HSP90, suppresses an increase in the interaction of HIF-1α with HSP90 in cancer cells [252]. Furthermore, combined treatment with IR and deguelin significantly overcomes radioresistance in lung cancer cells [252].

A gene therapy strategy targeting hypoxic cancer cells would be also a good option for enhancing radiosensitivity. A heterodimer with HIF-1α and HIF-1β binds directly to the hypoxia response element (HRE) present in the promoter regions of target genes. HIF-1/HRE-mediated transcriptional initiation has been suggested to facilitate induction of therapeutic gene expression in hypoxic regions of solid tumors [181]. In particular, the targeted expression of apoptosis-related genes using the HRE promoter in HIF-1-active malignant cancer cells will enable the resistance of hypoxic cells to IR to be overcome.

Novel drugs, such as antiangiogenic/vascular targeting agents, that target genetically stable vascular endothelial cells rather than unstable mutating tumor cells have been developed recently. Tumor angiogenesis enables division of the primary cancer cells by adequately delivering oxygen and nutrients, and targeting angiogenesis can enhance the efficacy of radiotherapy. Considerable preclinical evidence suggests the efficacy of tumor radiosensitization in combination with radiation and angiogenesis inhibition [253,254,255], and clinical trials of combinations of angiogenesis inhibitors with radiotherapy are underway. Novel drugs that cause vascular normalization or reduce tumor oxygen consumption will also be effective in terms of reducing tumor hypoxia. Additionally, direct evidence shows that downregulation of prostaglandins by Cox-2 inhibitors results in a reduction of angiogenesis increasing radiosensitivity [256], confirming the important role of angiogenesis in tumor radioresistance.

## 6. Perspectives and Conclusions

Cancer is the global leading cause of disease and injury, the incidence of which will increase due to the aging population. The number of patients receiving radiotherapy has been increasing steadily. Radiotherapy can benefit patients for whom surgery is not possible by significantly shrinking or destroying tumors. The exposure of tumors to IR triggers a variety of changes (either immediate or persistent), ranging from mild biochemical changes to several forms of death. The total dose and number of fractionation of IR can determine the degree and/or type of cellular damage. IR exerts anti-cancer activity by eliciting multiple DNA lesions, such as DSBs, SSBs, DNA crosslinks, and base modifications.

While radiation is effective against some types of tumors, other tumor types, such as pancreatic carcinoma and glioblastoma multiforme, are intrinsically resistant to conventional radiation therapy, making them more difficult to target. Several pathways determine the resistance of tumor cells to IR. These include DNA repair pathways, developmental pathways, the adaptive response pathway, adhesion pathways, the hypoxic pathway, and other survival pathways. Therefore, an urgent clinical need exists to develop radiosensitizers to overcome tumor radioresistance and thus enhance the effectiveness of radiotherapy. Combined chemotherapy and radiotherapy was clinically successful in the treatment of many tumor types. Clinical trials are investigating drugs that sensitize cancer cells to the effects of radiation, making them easier to destroy with radiation therapy. Meanwhile, protective drugs may facilitate the recovery of normal and healthy cells after exposure to radiation. Many current radiosensitization approaches target the DNA damage response. Overall, however, the clinical outcomes of these attempts using targeted drugs as radiosensitizers have generally remained disappointing. Therefore, novel radiosensitizing drugs should undergo more extensive and careful preclinical studies before being introduced in the clinic by increasing knowledge of their mechanisms of action.

To enhance the efficacy and cost-effectiveness of radiotherapy, the patient should also be treated with radiotherapy that is more fine-tuned. Advances in technology and biology have led to improvements in tumor delineation and targeting, resulting in improvement of tumor coverage and a reduction in normal tissue exposure. Good examples of progress in radiotherapy are technical advances such as image-guided radiotherapy (IGRT) and intensity-modulated radiation therapy (IMRT), which target the tumor more accurately, sparing healthy normal tissue. These allow physicians to shape radiotherapy very closely around a tumor from different directions, thereby reducing the impact of high radiation doses on nearby healthy cells such as the spinal cord or salivary glands, which, if damaged, would cause long-term problems. IMRT is a recently developed powerful therapeutic strategy that enables radiation oncologists to precisely control the distribution of radiation according to the overall shape of the tumor. Radiological imaging using computed tomography (CT) and magnetic resonance imaging (MRI) and multileaf collimators can enhance tumor specificity. With the help of these technologies, IMRT allows the delivery of precise radiation doses to tumors without increasing the adverse effects on surrounding normal tissue. In particular, simultaneous integrated boost IMRT (SIB-IMRT) enables the delivery of a booster dose of radiation to tumor cells with high efficiency and accuracy.
